# Slab narrowing in the Central Mediterranean: the Calabro-Ionian subduction zone as imaged by high resolution seismic tomography

**DOI:** 10.1038/s41598-018-23543-8

**Published:** 2018-03-26

**Authors:** L. Scarfì, G. Barberi, G. Barreca, F. Cannavò, I. Koulakov, D. Patanè

**Affiliations:** 1 Istituto Nazionale di Geofisica e Vulcanologia, Sezione di Catania - Osservatorio Etneo, Catania, Italy; 2 Dipartimento di Scienze Biologiche, Geologiche e Ambientali, Università di Catania, Sezione di Scienze della Terra, Catania, Italy; 3 Trofimuk Institute of Petroleum Geology and Geophysics, Novosibirsk, Russia; 4 Novosibirsk State University, Novosibirsk, Russia

## Abstract

A detailed 3D image of the Calabro-Ionian subduction system in the central Mediterranean was obtained by means of a seismic tomography, exploiting a large dataset of local earthquakes and computing algorithms able to build a dense grid of measure nodes. Results show that the slab is continuous below the southern sector of the Calabro-Peloritan Arc, but the deformation processes developing at its edges are leading to its progressive narrowing, influencing tectonics and magmatism at the surface, and with possible stress concentration in the tip zones. In the southwest, the deformation occurring at a free slab edge lead to propagation of a vertical lithospheric tear in the overriding plate, which extends along a NW-SE fault system (Aeolian-Tindari-Letojanni) up to about 30 km into the Ionian Sea; further southeast, the lithosphere appears only flexed and not broken yet. In the northeast, the slab seems to break progressively, parallel to the trench. Finally, northwest of Mt. Etna, the tomography highlights low V_P_ that can be related to an upwelling of deep mantle material likely flowing laterally through a window opened by the complete slab detachment.

## Introduction

**Figure 1:**
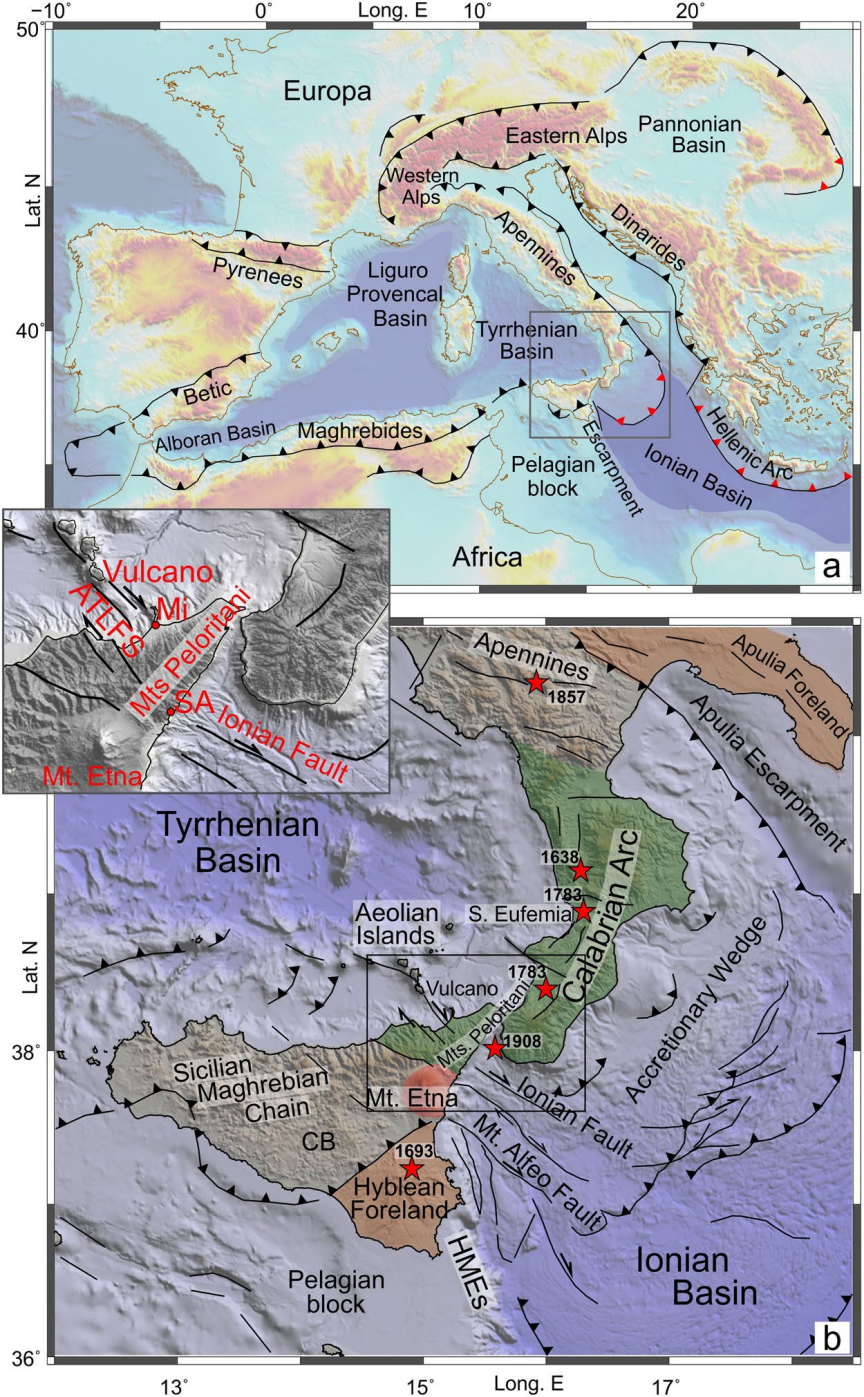
(**a**) Shaded-relief map of the Mediterranean realm showing the main orogenic belts, basins and subduction zones. Sawteeth point in the direction of subduction or underthrusting (in red where the subducting slab is considered to be continuous). (**b**) Main geological and tectonic features of the studied region (see text for references). Epicentres of the major earthquakes (M ≥ 7) of the last four centuries are shown as red stars (from https://emidius.mi.ingv.it/CPTI15-DBMI15/). Abbreviations are as follows: HMEs, Hyblean-Maltese Escarpment; CB, Caltanissetta Basin; ATLFS, Aeolian-Tindari-Letojanni Fault System; Mi, Milazzo; SA, Capo S. Alessio. Topography is from ref.^46^. The maps were created using QGIS (version 10.2.1; http://qgis.org) and Generic Mapping Tools (GMT version 4.5.6; http://gmt.soest.hawaii.edu). The figure was edited using Corel Draw Graphics Suite (version X5; http://www.corel.com).

Subduction processes have a great influence on the tectonic evolution and on the geologic architecture of a region, as well as on its seismicity and magmatism. Accordingly, their understanding and the zone where they take place are major topics in geological research. In the geodynamic context of the African and Eurasian plates convergence, the Mediterranean region provides an outstanding study opportunity, being the result of the long–lasting (35 Ma) NW-dipping subduction of the ancient Ionian oceanic lithosphere. The present-day tectonic framework of the central-western Mediterranean arises from the evolution of this subduction process, which has been characterized by the rapid SE-ward rolling-back of the Ionian slab and by the opening of large back-arc extensional basins (i.e., Liguro-Provençal, Algerian, Alboran, and Tyrrhenian basins, Fig. 1a). This deformation produced lateral migration and relative rotation of orogenic wedges (e.g. refs^1,2^). Moreover, the slab retreating and trench shifting towards the foreland was accommodated by lateral tearing which split adjacent segments of the subducting lithosphere; as a result, the active portion of the system has become progressively narrower^3,4^. Currently, active subduction residue of the ancient and wide trench zone is found beneath the Calabro-Peloritan Arc (Fig. 1), which is one of the sectors of maximum distortion of the orogen surrounding the Mediterranean, linking the Sicilian-Maghrebian Chain and the southern Apennines (Fig. 1b). This area, which is surrounded by active volcanoes (Etna, Aelolian Islands, Vesuvio), experienced several destructive earthquakes with estimated magnitudes of about 7 or higher (e.g., March 1638, February and March 1783, December 1908; see ref.^5^).

Much effort has been made by previous investigators to understand the evolutionary phases and to describe the deformation mechanisms that characterize the Calabro-Ionian subduction system (e.g. refs^4,6,7^). There is a general consensus that the slab is still continuous in-depth along a quite narrow segment of the Calabro-Peloritan Arc; however, details about the structure and the processes currently occurring and how they influence the tectonic regime and magmatism of the region are still elements inadequately constrained and matter of discussion. In particular, a number of studies have dealt, often with discordant conclusions (e.g. refs^8–11^), with the slab edges, which are tectonically important features, and with the way they deform. In recent years, interpretation of seismic profiles along the Ionian Sea have allowed to reconstruct the shallow part of the Calabrian accretionary wedge and of its southernmost edge (see refs^9,11,12^). Several km-long structural lineaments have been identified, some of which have been interpreted as the shallow expression of lithospheric-scale tear faults bounding the subducting plate (STEP - Subduction Tear Edge Propagator faults, according to ref.^13^); however, an in-depth lithospheric image of the subduction system is lacking. Detailed knowledge of the shape of the slab is critical element for modeling the subduction system evolution and the related tectonic processes.

In this regard, seismic tomography is the most effective method to explore the deep structure of subduction zones. Seismicity can reveal the plate interactions and map the Wadati-Benioff zone; moreover, the existence of positive seismic velocity anomalies in the upper mantle can be interpreted as the effect of cold oceanic lithosphere that penetrates a warmer and lower velocity asthenosphere, whereas, low velocity regions are preferably associated with partially molten rocks (e.g. refs^14,15^).

In this paper, we focus on improving the image of the Calabro-Ionian subduction system, by means of a local earthquake tomography (LET), in order to better understand the geodynamics and tectonics of the whole region. For this goal, we considered a large dataset of about 20,100 earthquakes, encompassing both shallow and deep seismic events located in southern Italy and in the central Mediterranean, which was exploited to obtain a reliable 3D velocity structure and accurate hypocentre locations. We took advantage of recently developed computing algorithms, which are able to build an adaptive grid of measure nodes according the seismic rays density (LOTOS by ref.^16^) and to improve the relative position of clustered events (tomoDDPS by ref.^17^). As a result, we were able to investigate the large scale tectonic structures in unprecedented detail with respect to previous comparable tomographic studies (e.g. refs^7,18^).

## Results

In this section, we illustrate the results of the combined analysis of earthquake relocation and seismic tomography.

**Figure 2:**
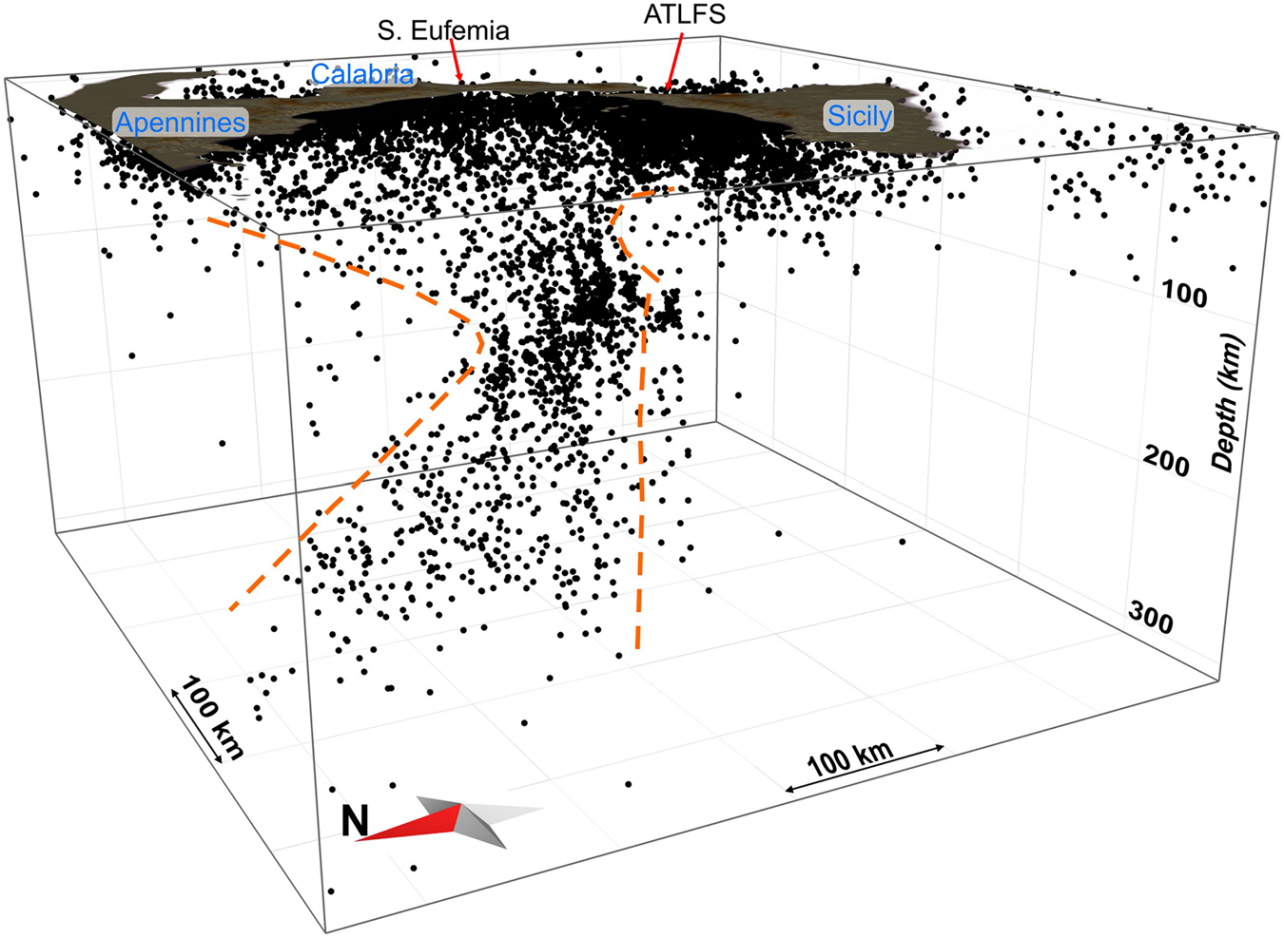
3D view of the localised earthquakes. The orange dashed lines highlight lateral slab boundaries arising from this study (see Results). The plot was created using Move (version 2017.2; https://www.mve.com) and Corel Draw Graphics Suite (version X5; http://www.corel.com).

**Figure 3:**
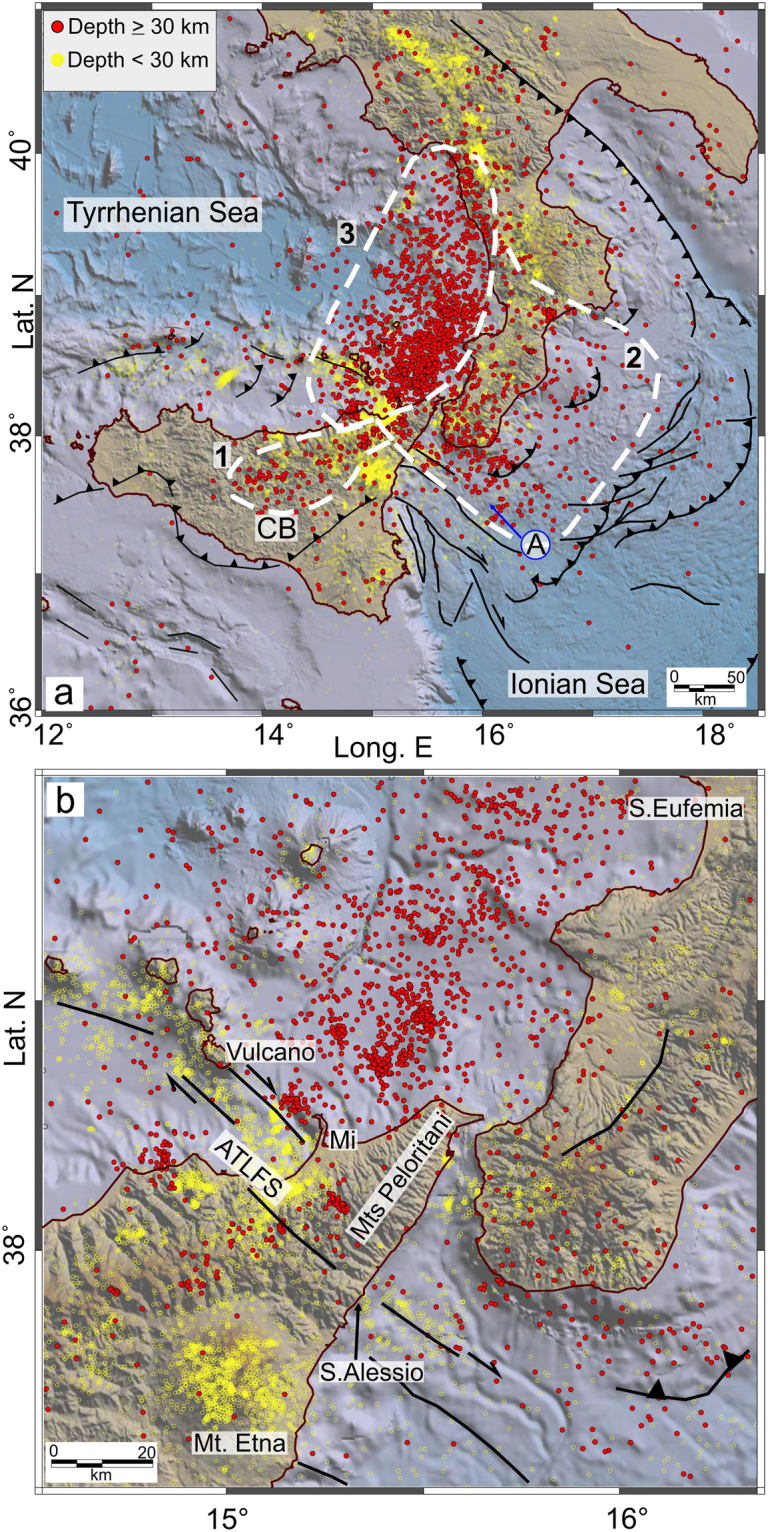
Final event locations. Numbers and white dashed lines denote the identified seismogenic domains (see Results for further details). The circled letter A indicates features described in the text. The main tectonic structures are also shown. Topography is from ref.^46^. The maps were created using QGIS (version 10.2.1; http://qgis.org) and Generic Mapping Tools (GMT version 4.5.6; http://gmt.soest.hawaii.edu). The figure was edited using Corel Draw Graphics Suite (version X5; http://www.corel.com).

**Figure 4:**
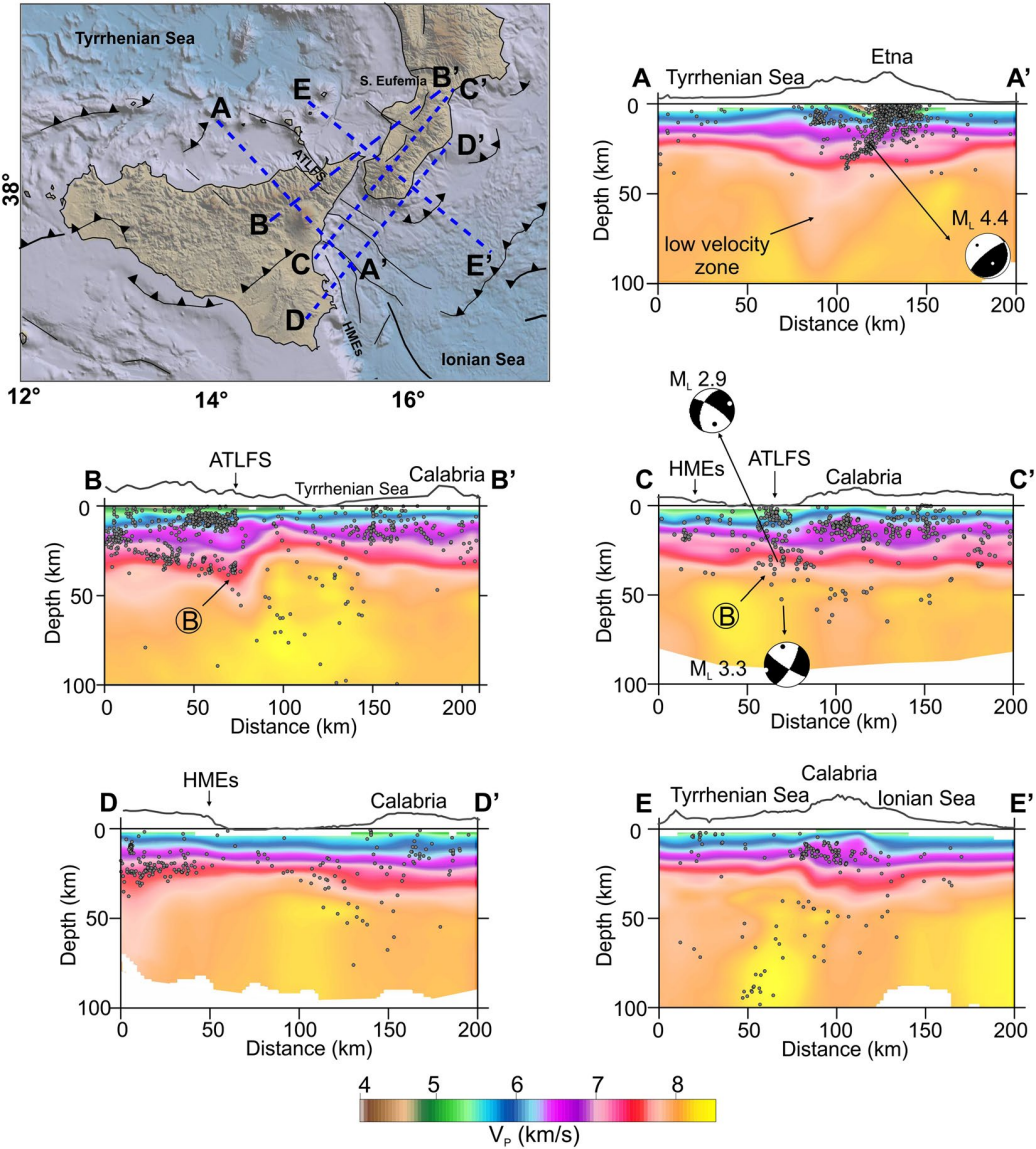
Vertical sections through the V_P_ model, in km/s. Relocated earthquakes, within ±5 km from the cross-section lines, are plotted as grey dots. Focal solutions are by refs^20,30^. Topographic profiles at the top of the sections are only indicative of the geographic location. Circled letters indicate features described in the text. The traces of the sections (AA’, BB’, …) are reported in the sketch map. Topography is from ref.^46^. The figure was obtained using the software Surfer (version 10.7.972; http://www.goldensoftware.com/products/surfer) and QGIS (version 10.2.1; http://qgis.org). The plot was edited using Corel Draw Graphics Suite (version X5; http://www.corel.com).

A 3D view of the relocated earthquakes is shown in Fig. 2. With the aim of better understanding the patterns of the crustal structures and the slab-related features, epicentres are displayed in Fig. 3 in relation to their depth: i.e., shallow (<30 km) and deeper (≥30 km) as yellow and red filled circles, respectively. While shallow seismicity shows a more complicated pattern according to the main crustal structures, the distribution of earthquakes with depths >30 km reveals that most of them are gathered in three seismogenic zones, identifiable on the basis of the event concentration and geological domains. The first one is pinpointed by a NE-SW event alignment that extends along central-eastern Sicily from the Caltanissetta Basin to the northeastern limit of the Sicilian-Maghrebian Chain (southwest of the Peloritani Mountains; n. 1 in Fig. 3a). In cross-section view (Fig. 4AA’), the related hypocentres and the lateral discontinuities in the velocity model suggest NW-dipping tectonic structures in the 15–35 km depth range, resembling deep-seated thrusting. The V_P_ model also discloses low values located NW of the Etna volcano, down to about 80 km of depth.

**Figure 5:**
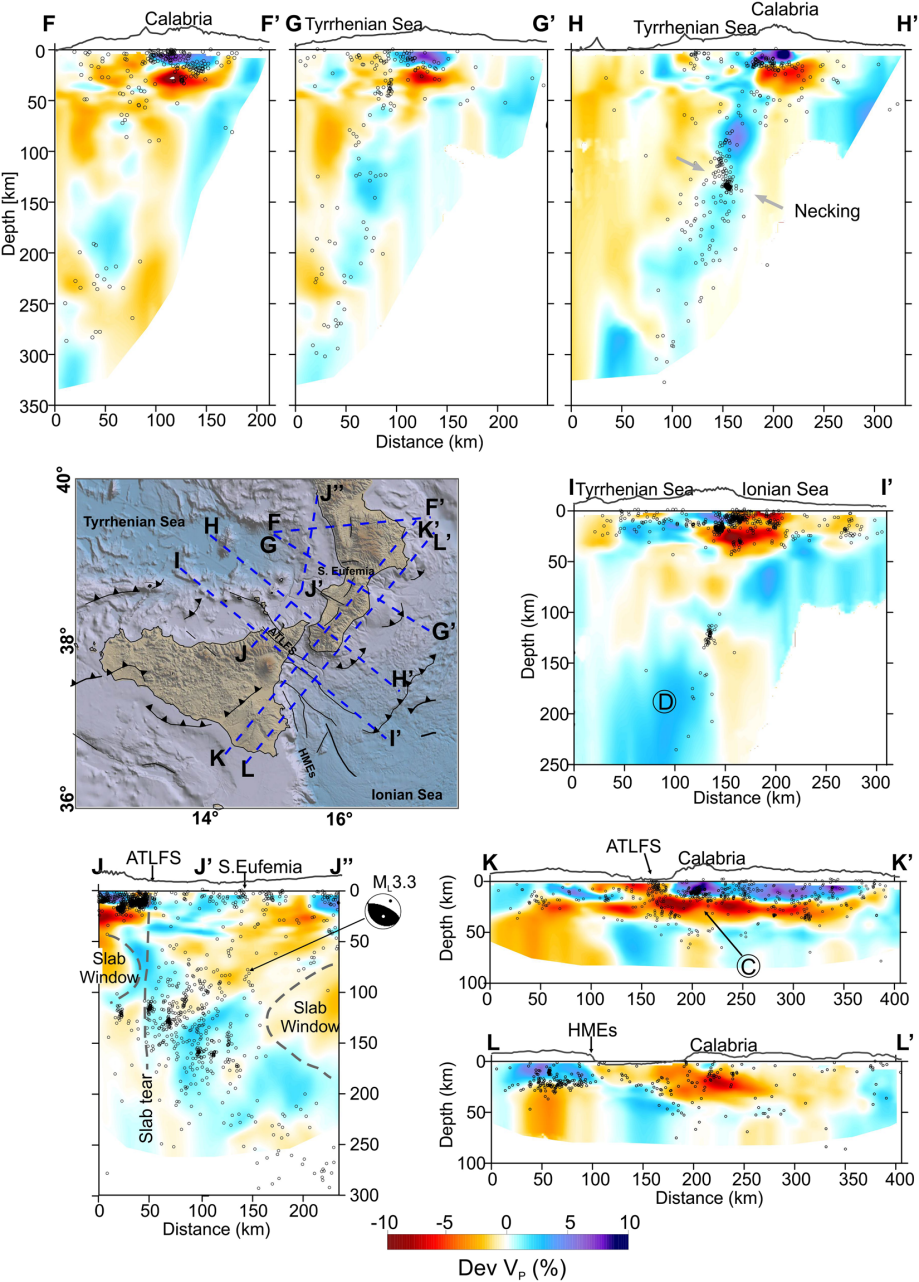
Vertical sections through the V_P_ model, as perturbations (%) relative to the initial velocity model. Relocated earthquakes (grey dots) are within ±5 km from the cross-section lines. Focal solution is from ref.^20^. Circled letters indicate features described in the text. Topographic profiles at the top of the sections are only indicative of the geographic location. Topography is from ref.^46^. The figure was obtained using the software Surfer (version 10.7.972; http://www.goldensoftware.com/products/surfer) and QGIS (version 10.2.1; http://qgis.org). The plot was edited using Corel Draw Graphics Suite (version X5; http://www.corel.com).

**Figure 6:**
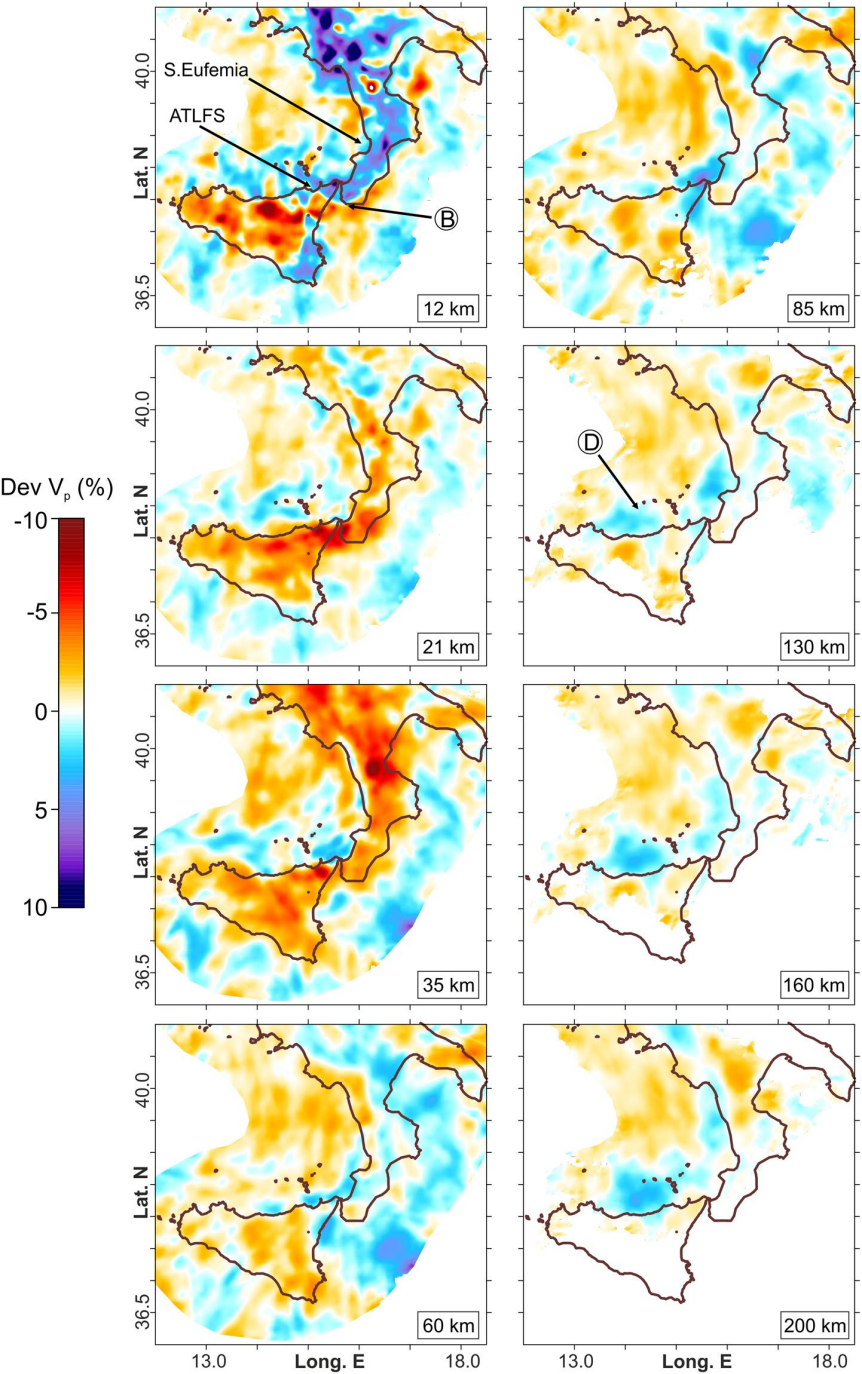
V_P_ perturbations (%) relative to the initial velocity model for the most representative layers resulting from the 3D inversion. Circled letters indicate features described in the text. The figure was generated using the software Surfer (version 10.7.972; http://www.goldensoftware.com/products/surfer).

A second zone is identified by the earthquakes located between continental Calabria and the Ionian Sea. In this sector, most of the epicentres depict a wide seismogenic block (n. 2 in Fig. 3a), roughly between the S. Eufemia Gulf to the north and the Sicilian offshore, about 20 km off the southern coast of Calabria. While along its northern limit the events are quite scattered, in that to the south they form a sharp alignment NW-SE striking (A in Fig. 3). Along this direction, moving toward the Sicilian coast, the deeper seismicity (>30 km) links with the shallower earthquake clusters that are located between the Aeolian Islands and Capo S. Alessio and that characterize the fault system known as Aeolian Tindari Letojanni Fault System (ATLFS; Fig. 3b). As a whole, between Vulcano and the Ionian Sea, the events are vertically clustered down to 40–50 km of depth (B in Figs 4BB’,CC’ and 5KK’) and outline a narrow seismic zone which is also evidenced by a sharp discontinuity in the V_P_ model (Figs 4 and 6 at 12–60 km). This pattern is found up to ~30–40 km off the Sicilian coast alongside the ATLFS; further to the southeast, seismicity seems to delineate a gently sloped seismic layer which deepens from the zone where main offshore faults are mapped (e.g., the Mt. Alfeo fault system, see ref.^10^) to a deeper seismic level beneath the Calabrian accretionary wedge (~ 40–50 km of depth; Figs 4DD’ and 5LL’). Beneath southern Calabria, earthquake distribution points out two seismogenic layers that appear separated by a zone of low velocity anomaly (C in Fig. 5KK’).

Events deeper than 70 km are located in the SE sector of the Tyrrhenian Sea, according to a trend following the western Calabrian coastline (3^rd^ seismic zone; n. 3 in Fig. 3a). Here, the in-depth earthquake distribution outlines an “hourglass”-shaped feature (Figs 2 and 5JJ”); in particular, this seismicity, which delineates the Wadati-Benioff plane, results continuous only between the northern tip of Sicily and the S. Eufemia Gulf. Northward, hypocentres point to a large “V-shaped” aseismic zone with the vertex around 130 km of depth below the S. Eufemia Gulf, which progressively enlarges up to the northern end of Calabria; to the south, on the contrary, intermediate and deep events depict a sharper limit around the ATLFS. The high-velocity anomalies revealing the subducting lithosphere show a continuous vertical profile (though with hints of necking at 100–150 km of depth) according to the hypocenter distribution, i.e., between Milazzo and the S. Eufemia Gulf. Further north, these anomalies, which follow the coastline, appear vertically fragmented (Figs 5FF’–HH’ and 6). Southwestward, the tomography indicates a clear segmentation of the slab in the correspondence of the ATLFS; in particular, a separated high V_P_ anomaly consistent with a subducted lithosphere emerges to the west of the ATLFS, from 100–150 km of depth downward (D in Figs 5II’ and 6). It is characterized by scarce seismicity, which occurs only in its upper part and which seems slightly shifted (~15 km) toward NW with respect to the events located to the east of the ATLFS at the same depth.

Finally, the tomographic images also well reflect the variations of the Moho depth, showing a thinning of the slower velocity and shallower layers corresponding to the oceanic crust zones (e.g., see Fig. 4).

## Discussion

The tomographic images and the earthquake locations presented here are in agreement with the results of previous studies (e.g. refs^7,18^) about the overall structure of the descending Ionian slab: it is continuous at depth only below the southern Calabro-Peloritan Arc, where its curvature is increased^19^, specifically between the ATLFS and S. Eufemia Gulf; it is very steep (70°), at least down to 200 km of depth. However, processing of new data has allowed us to better image the 3D architecture of the subduction zone, which proves to be strongly controlled by the deformation processes currently developing at its edges and in particular by large tears leading to progressive narrowing and necking of the slab.

In the northeast, the slab is dying out along a horizontal breakoff that propagates in a scissor-type mode. This mechanism can be principally inferred from the occurrence of a triangular-shaped aseismic zone (Figs 2 and 5JJ”) that has its tip at 130 km of depth below the S. Eufemia Gulf and becomes wider to the north. The existence of this feature is also supported by the high-velocity anomaly, the signature of the slab, which appears fragmented in northern Calabria and almost entirely absent in the shallow layers (Fig. 5FF’). The compressive kinematics around the tip of the triangular-shaped slab window, inferred from some available focal solutions (refs^20,21^; see Fig. 5JJ”), is consistent with the findings of ref.^22^, pointing to high interplate frictional force at the edge of the lateral breakoff zone within the Vrancea slab.

In the southwest, there is evidence for a “free” slab edge, produced by a combination of vertical and horizontal tearing which may be hypothesized according to the model by ref.^23^, leading to slab breakoff and retreat. In fact, velocity anomalies clearly pinpoint the existence, west of Milazzo and from about 100 km of depth downward, of a slab fragment that is completely detached at shallow levels (D in Figs 5 and 6); this implies a horizontal breakoff which has led to the formation of a slab window between 50 and 100 km of depth below the western portion of the Peloritani range (Fig. 5JJ”; see also ref.^24^). Vertical breakoff can be deduced by the substantial absence of deep seismicity west of Milazzo and by the deep lateral velocity discontinuity. This tear has a clear expression at the crustal level by vertically clustered events (down to 40 km of depth) and discontinuities in the V_P_ model which can be observed in the correspondence of the ATLFS and its prosecution in the Ionian offshore (A in Fig. 3, B in Figs 4 and 6). These features depict a narrow deformation corridor, perpendicular to the slab hinge, that is characterised by evident right transtensional kinematics on NW-SE tectonic structures as discovered by geological and geodetic observations (e.g. refs^25–28^) and confirmed by NW-SE dextral or oblique slip focal mechanisms of events deep down to about 70 km^29,30^ (see focal solutions in Fig. 4CC’). Such a deformation regime is expected in case of propagation at the surface of a vertical tearing fault, which characterizes the edge of a retreating and still continuous slab. However, it must be noticed that this pattern featuring a vertical sub-lithospheric tear is observed up to 30–40 km off Capo S. Alessio on the Sicilian coast. South and southeastward, hypocentres and V_P_ velocity distribution instead suggest an array of tectonic structures, building a larger deformation zone that delimits laterally the Hyblean (continental) and the subducting Ionian (oceanic) lithospheres (Fig. 5LL’). This deformation zone lies atop a large flexure of the Hyblean-Ionian lithosphere probably produced by the load of a grown orogen beneath Calabria (the Apennines-Maghrebian Chain). Orogenic piling below continental Calabria is clearly suggested by the tomography sections (e.g. Fig. 5KK’), which display a 15–20 km thick low velocity layer sandwiched between two high- velocity bodies, the European at the top and the Ionian oceanic crust below. As observed at a number of subduction zones (e.g. refs^31,32^), this layer may be interpreted as being constituted by weak material (soft sediments) scraped from the subducted oceanic crust and accreted in the inner portion of the Calabrian accretionary wedge.

Another remarkable feature is the large low velocity volume found NW of Mt. Etna at the southern slab boundary. Its extension down to 80 km of depth could indicate upwelling of warm and less dense mantle material to shallower depth. Indeed, the existence of slab tears may create a window through which asthenospheric mantle can flow^33,34^ between sectors of material with different densities. These mechanisms may explain changes in seismic anisotropy and anomalous melting patterns in surface volcanism (see ref.^35^). In particular, along the Calabro-Ionian subduction region, the direction of the SKS fast wave polarization, usually related to mantle flow, presents an abrupt rotation from trench-parallel to perpendicular near the southwestern slab edge (see refs^36,37^). Moreover, the geochemical signatures of fluid enrichment and magma mixing in Mt. Etna lavas have been considered a consequence of the progressive opening of a slab window through time (e.g., refs^38–41^). Deep seated thrusting beneath Mt. Etna (Fig. 4AA’, see also ref.^42^) could be seen as the consequence of the wedging of the low velocity body in the area^41^.

Earthquakes and V_P_ patterns also give hints about the current structure of the subduction zone where the slab is still continuous; in particular, in the section HH’ (see Fig. 5) an incipient slab necking at 130 km of depth, highlighted by earthquake clusters, is shown.

## Conclusions

**Figure 7:**
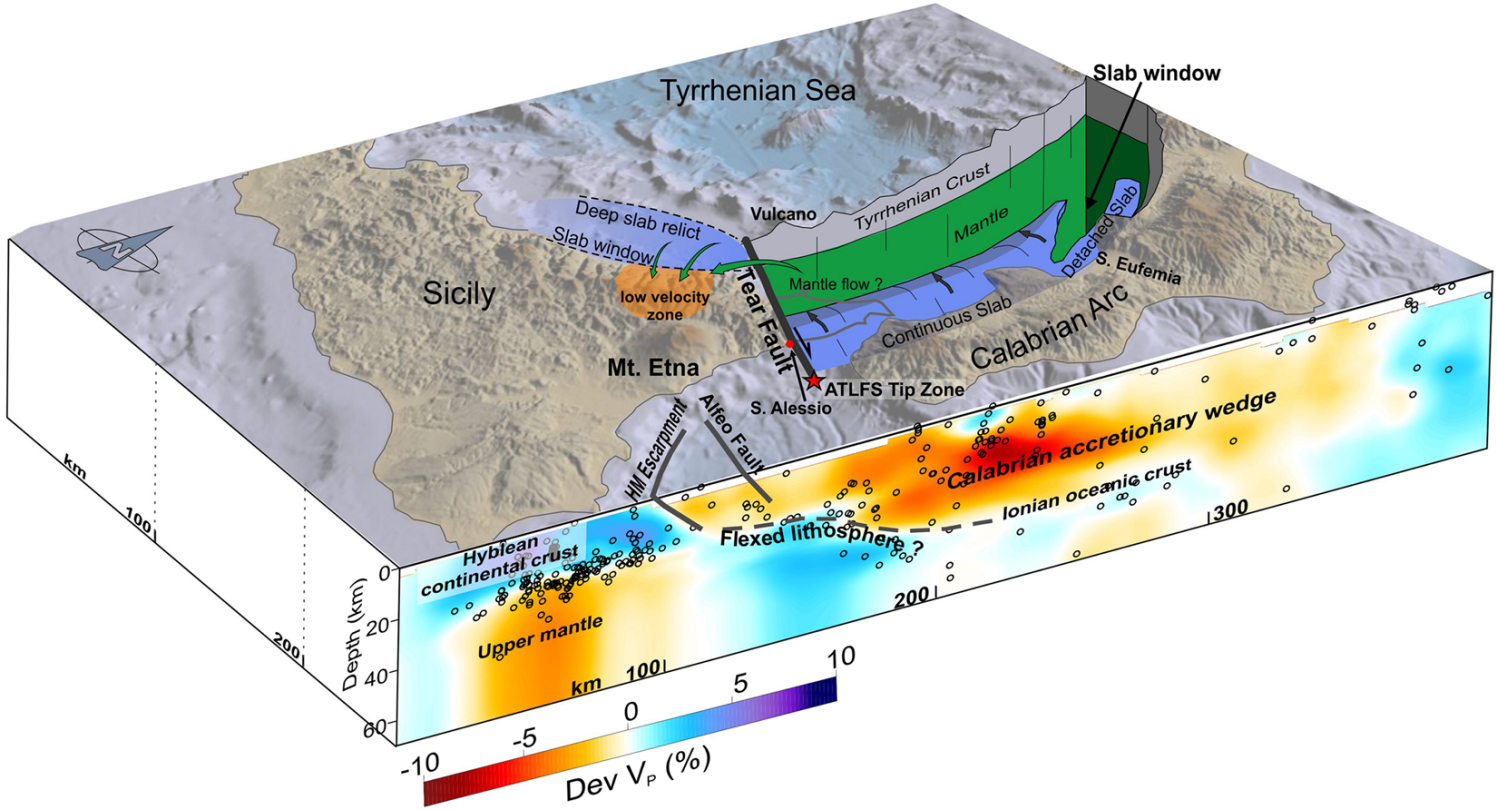
3D sketch summarizing the main features deriving from this study. Topography is from ref.^46^. The figure was obtained using the software Move (version 2017.2; https://www.mve.com) and Corel Draw Graphics Suite (version X5; http://www.corel.com).

The key findings from the tomographic images and accurate event locations have allowed us to better outline the structure and the deformation mechanisms which characterize the Calabro-Ionian subduction zone and which have significant implications for the surface tectonic and magmatic processes. The main findings are summarized in Fig. 7.

An important aspect revealed in the study is the dynamics occurring at the slab edges. In particular, along its southwestern boundary, where the sinking and the retrograde movement of the slab have caused its segmentation, the deformation is expressed by a combination of a lithospheric vertical tear and a horizontal slab breakoff. The vertical lithospheric tear propagates in the upper plate along the NW-SE oriented Aeolian Tindari Letojanni Fault System up to its tip zone, which has been identified about 30 km off Capo S. Alessio in the Ionian Sea. Moving toward the SE (i.e. along the Ionian Fault) no evidence for lithospheric tearing were found; the Hyblean-Ionian lithosphere appears only flexed and not broken yet. Previously mapped faults in the Ionian offshore^9–11^ lie atop the flexed zone and seem to accommodate this kind of deformation at the shallow crustal level in a context of incipient slab edge bending and tearing.

On the northern side, the slab has been progressively broken parallel to the trench and the horizontal tear may still propagate southwards. Horizontal tearing affecting both the sides of the slab results into a narrowing of the subduction system with consequent stress concentration at the tip zones and enhanced subsidence due to the gravitational pull along the intact segment of the slab^19^.

This overall image can be confirmed by the topographic profile along the Calabro-Peloritan Arc, which shows a relatively depressed region corresponding to the area overlying the intact slab and highs at its edges^43^ (i.e. west and southwest of the Mts Peloritani and northeast of S. Eufemia). This matches the scheme postulated by ref.^19^.

A lot of the analysed seismicity appears to be located near the slab hinge; in particular it is concentrated along the former backstop of the overriding European crust rather than, as expected, at the subduction interface where, conversely, events appear quite sparse. Deeper earthquakes occur within the high-velocity layer (the descending slab) highlighting the deformation of the Hyblean-Ionian lithosphere beneath the Calabrian crust. In the accretionary wedge, likely made of weak material scraped off the subducted Ionian oceanic crust, seismicity is quite low.

In conclusion, slab narrowing by the horizontal breakoff and its necking by gravitational pull are factors that must be considered in assessing the future seismic potential of the Calabro-Ionian subduction system, in addition to its still rupturing southern edge.

## Data and Methods

Initial hypocentral parameters and seismic phases used in the study have been taken from the national and local catalogues managed by the Istituto Nazionale di Geofisica e Vulcanologia, containing systematic observations of the seismic activity in Italy and Central Mediterranean since the 1980s (see http://istituto.ingv.it/index.php/it/archivi-e-banche-dati). From these databases, we selected earthquakes recorded between 1981 and 2014 within the area of interest (see rectangle in Fig. 1a), with at least 7 observations (minimum 5 P and 1 S readings) and maximum azimuthal GAP of 270°. We were more restrictive in the Mount Etna zone, where the events above sea level and with magnitude less than 2.5 were discarded, in order to exclude most of the seismicity of the complex shallower structure of the volcano, which is beyond the scope of this work. The final dataset includes about 20,100 events with magnitude between 0.3 and 5.8, whose hypocentral distribution provides initial evidence of a good ray-coverage of the earth volume beneath Calabria and Sicily (see Supplementary Fig. S1).

### Local earthquake tomography algorithm

Seismic velocity modeling (V_P_, V_S_, and V_P_/V_S_) was carried out by the program LOTOS^16^, by inverting a total of 236,048 P and 104,616 S arrival times recorded at 770 stations. Before performing the tomography, it has been necessary to define a set of parameters and in particular a preliminary guess for the 1D seismic velocities, which was derived from the model optimized for the same area by ref.^44^. The average value of V_P_/V_S_ ratio reported in the literature (1.75) was checked by a Wadati diagram^45^.

The grid nodes were established exploiting an important feature of the LOTOS code, which automatically sets up the inversion mesh according to the distribution of the seismic rays. Specifically, given a regular grid step, which should be smaller than the presumed size of the anomalies, the software is able to not install any node in the case of absence of rays, whereas in areas of higher ray number, it can increase the grid density up to the maximum value (in our case, 8 and 3 km for the horizontal and vertical direction, respectively). The inversion solution is further controlled by smoothing parameters which reduce the difference in the final values of neighboring nodes. The ability to build such a dense (quasi continuous) mesh is fundamental for a “local earthquake tomography” in order to reduce the bias of the resulting models due to the grid configuration (i.e. too large spacing between nodes to suitably image earth heterogeneities). Fine-tuning of the inversion was then obtained by discarding events with less than 12 observations and arrival-times with residuals of more than 1.5 s for P and 2.5 s for S rays.

### Synthetic tests

The accuracy of the final 3D results was assessed by tests with numerical models. The procedure consists of calculating theoretical travel times of the seismic rays for an idealized velocity model (target model). The synthetic data are then inverted using the same starting model and control parameters as for the real data. By comparing the results of this inversion with the target model, one can detect unrestored zones that correspond to areas where tomographic resolution is low.

In the first series of tests, we considered traditional checkerboard models composed of blocks with alternating positive and negative anomalies (see Supplementary Figs S2 and S3). We tested working with 3D anomalies of different size and, to simulate picking uncertainty, perturbed the travel times with random noise having an average deviation of 0.1 s for both P and S-wave data. Other tests were performed with more realistic configurations of synthetic anomalies, which better simulate the complexity of the tectonic structures investigated (see Supplementary Figs S4 and S5).

The results of these tests clearly indicate that the V_P_ anomalies are adequately recovered in all the areas where the experimental tomographic images are interpreted. At shallower depths (down to about 20–30 km) we can robustly recover small anomalies (10–15 km of size). Increasing the depth, larger features can be recovered, as the seismic ray coverage gradually reduces, and limitations in resolution emerge in some parts of the study area. However, the imaged structures and the slab are well resolved, at least down to about 200 km.

See the Supplementary material for a complete description of the synthetic tests.

### Event location

Earthquake locations of the whole dataset were further enhanced by the tomoDDPS algorithm^17^, which has the advantage of using a combination of both absolute and differential arrival time readings. In case of clustered events, additional information is used to improve the relative locations: i.e., since the uncertainty of the velocity model along the station-hypocenter path is the same for all the earthquakes, the travel-time differences found at a common station can be attributed to the spatial offset between the events. This procedure produced better clustering and further reduced the residuals (RMS) of about 55%, with an average of 0.3 sec.

### Data availability

Most of the earthquake parameters are available on the INGV institutional website (http://istituto.ingv.it/index.php/it/archivi-e-banche-dati). The LOTOS code can be downloaded from http://www.ivan-art.com/science/LOTOS. The tomoDDPS algorithm was provided by H. Zhang (zhang11@ustc.edu.cn). Data not available on these websites can be provided by the corresponding author on reasonable request.

## Electronic supplementary material

Supplementary Information
