# Supplementary material for: Slab narrowing in the Central Mediterranean: the Calabro-Ionian subduction zone as imaged by high resolution seismic tomography
Source: Sci Rep. 2018 Mar 26;8:5178. doi: 10.1038/s41598-018-23543-8 (PMC5980090; doi:10.1038/s41598-018-23543-8)
Supplement: Supplementary file 1 — Supplementary Information [file 41598_2018_23543_MOESM1_ESM.pdf]

Slab narrowing in the Central Mediterranean: the Calabro-Ionian subduction zone as imaged by high resolution seismic tomography

L. Scarfì[1,*], G. Barberi[1], G. Barreca[2], F. Cannavò[1], I. Koulakov[3, 4], D. Patanè[1]

1) Istituto Nazionale di Geofisica e Vulcanologia, Sezione di Catania - Osservatorio Etneo, Catania, Italy
2) Dipartimento di Scienze Biologiche, Geologiche e Ambientali, Università di Catania, Sezione di Scienze della Terra, Italy
3) Trofimuk Institute of Petroleum Geology and Geophysics, Novosibirsk, Russia
4) Novosibirsk State University, Russia

*Corresponding author: luciano.scarfi@ingv.it

This supplementary document illustrates the seismic ray coverage in the area studied by the seismic tomography and describes the reliability analysis performed to assess the robustness of the solutions.

P-wave ray path

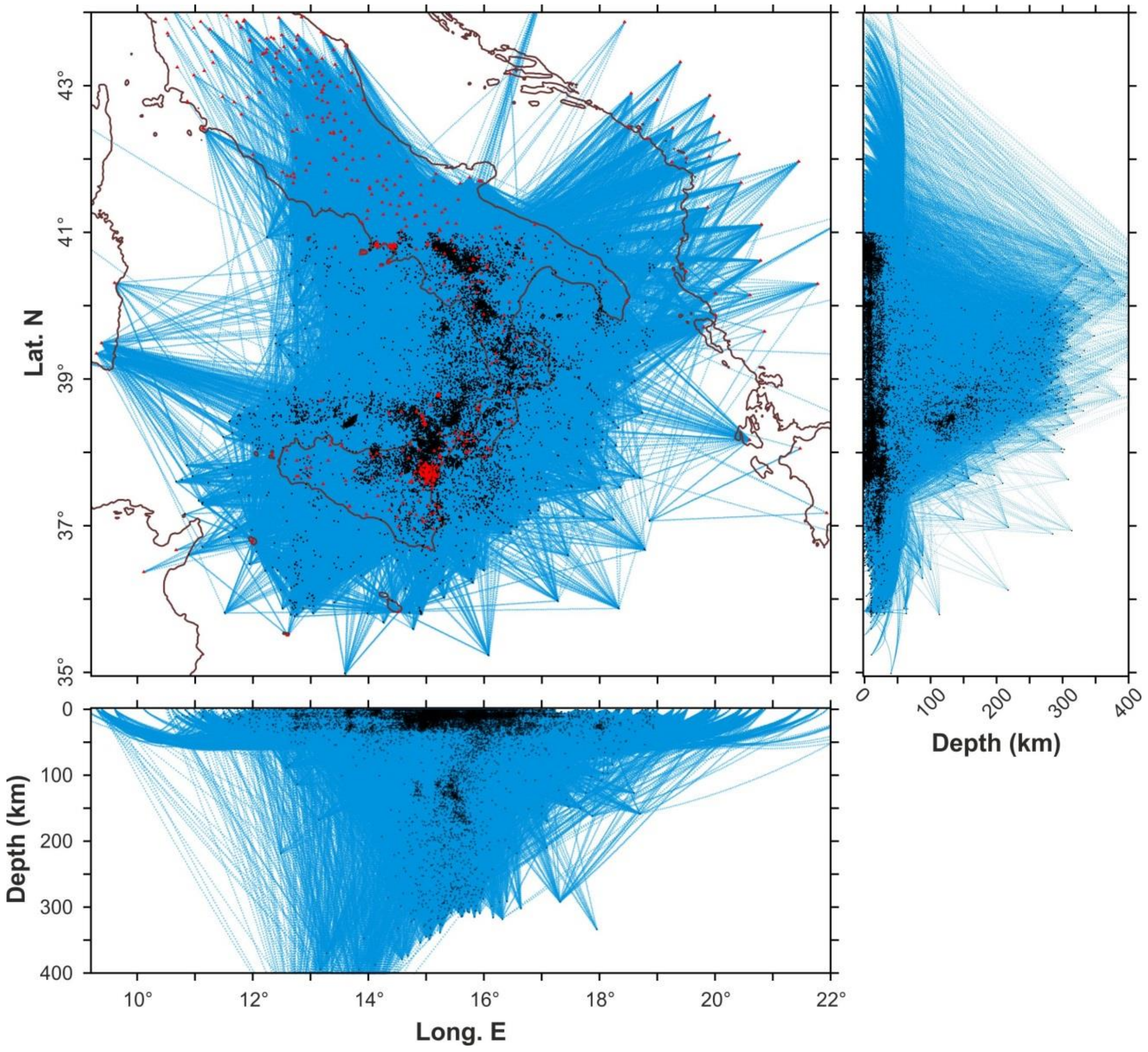

Figure S1. P-wave ray paths traced as straight lines in map, NS and WE sections. Red triangles and black points indicate stations and earthquakes, respectively. The figure was obtained using the software Surfer (version 10.7.972; http://www.goldensoftware.com/products/surfer) and Corel Draw Graphics Suite (version X5; http://www.corel.com).

Synthetic tests

To assess the resolution limitations of our tomography, we have performed a series of synthetic tests, which consist in reproducing an idealized velocity model (target) by inverting theoretical travel times associated with it. The synthetic model is defined as superposition of a “known” 1D velocity model and 3D anomalies of the P and S-wave velocities. The synthetic travel times are computed using the bending ray tracing code for the actual source-receiver pairs corresponding to the final iteration of the experimental data inversion. In our case, to simulate picking uncertainty, these times were perturbed with random noise having the average deviation of 0.1 s for both P and S-wave data.

The recovering procedure uses the same workflow and control parameters as in the case of the experimental data processing. In particular, the calculations start with the location of the seismic sources using the initial 1D velocity model. This step is very important to assess adequately the realistic resolution capacity related to the trade-off between source and velocity parameters. By comparing the results of the inversion with the target model, one can detect unrestored zones, which correspond to areas where tomographic resolution is low.

In the first series of tests, we considered traditional checkerboard models, which are composed of blocks with alternating positive and negative anomalies. The amplitude of the anomalies was set to ±7%, which represents the average range of variations in the actual velocity model. With depth, these anomalies change of sign every 60 km. Figure S2 shows the results for some representative layers of three models, having anomalies with lateral sizes of 30, 40 and 60 km, respectively. The recovered models indicate that larger features can be recovered increasing the depth. The anomalies are robustly resolved in all parts of the study area down to 30 km of depth; at 60 km, the resolution is sufficiently achieved in most zones, whereas at 120 km depth the anomalies are smeared but still recognizable beneath Sicily, Calabria and along the Tyrrhenian coast. Still deeper, the ray-coverage further reduces but remains effective in the area affected by the slab.

A similar test was performed with checkerboard anomalies (50x50 km) defined in some of the vertical sections used for presenting the main results (Figure S3). Again the test shows limitations in vertical resolution in some parts of the study area where there are not enough data; nevertheless, the anomalies are adequately recovered in all the zones where the experimental tomographic images are interpreted.

The cases with realistic configurations of synthetic anomalies are considered in Figure S4. The 3D velocity anomalies are set in a series of polygonal prisms defined in some depth intervals. To define this model, we use the shapes of anomalies obtained from experimental data inversion at different depths. The inversion results indicate that the most important anomalies observed in our main

experimental model appear to be reliable. At the shallowest depths, we robustly recover both large positive and negative areas and a series of small anomalies of 10-15 km size. At 30 km depth, we can reconstruct anomalies that can be associated with variations of the Moho depth. At 80 km, we can clearly resolve a zone of the slab initiation. To explore carefully the quality of the slab reconstruction below 100 km of depth, we considered two different synthetic models. In one of them, the slab is represented by a series of separated blocks (4th column in Figure S4), whereas in the second one, the slab is continuous (5th column). When comparing the recovery results, we can conclude that the tomography is capable to resolve the gaps; in the case of continuous slab, a slight loss of the amplitude of the anomaly can be noted in northern Calabria.

In Figure S5, we present a synthetic model used to explore the capacity to resolve the slab shape in vertical section. We see that the slab is well resolved down to about 200 km. In the deeper part, it is still visible, but slightly lose the amplitude.

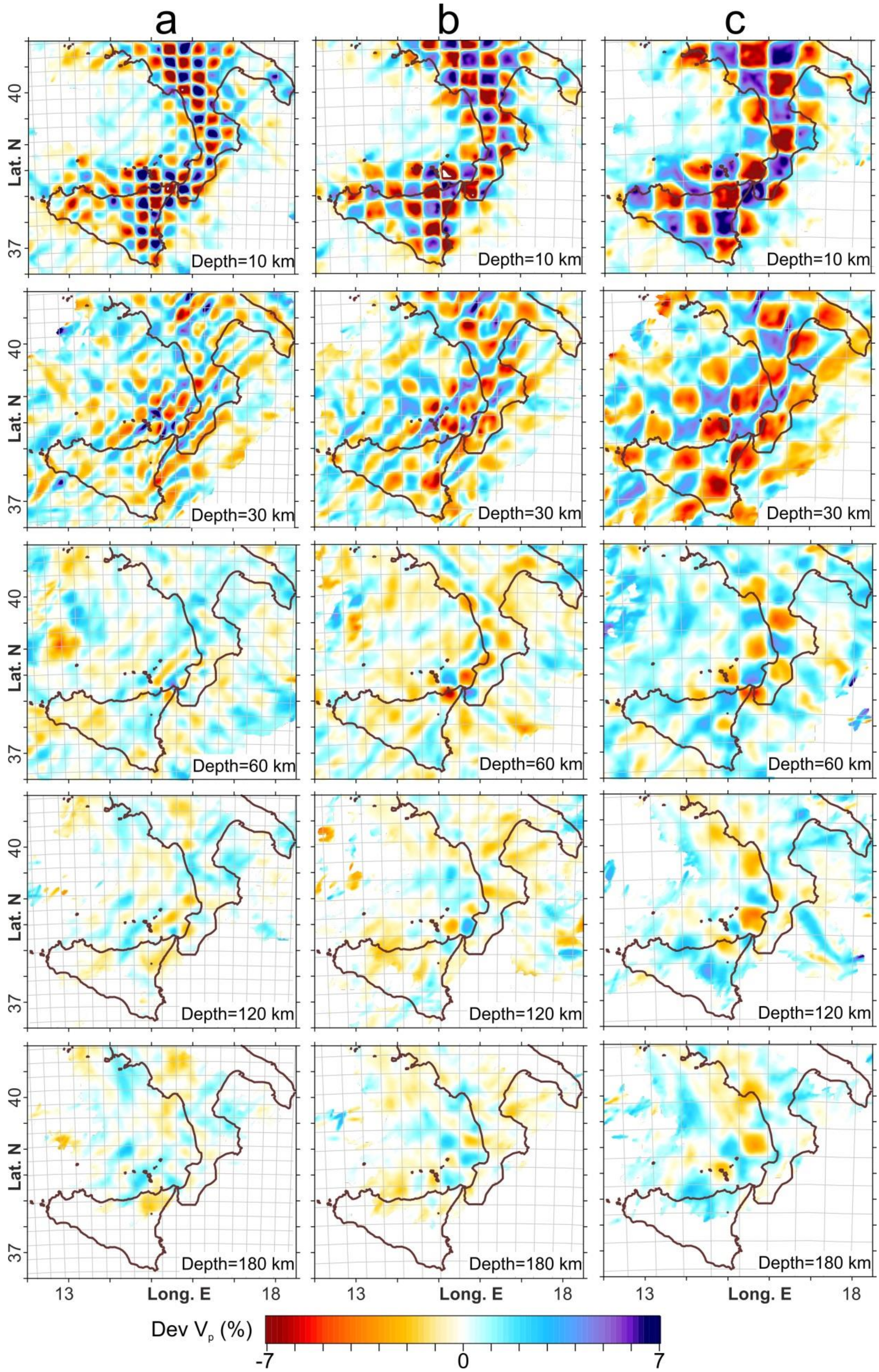

Figure S2. Results for some representative layers of three checkerboard models having anomalies with lateral sizes of 30 (column a), 40 (column b) and 60 km (column c). Grey thin lines highlight

the configurations of the synthetic anomalies. The figure was obtained using the software Surfer (version 10.7.972; http://www.goldensoftware.com/products/surfer) and Corel Draw Graphics Suite (version X5; http://www.corel.com).

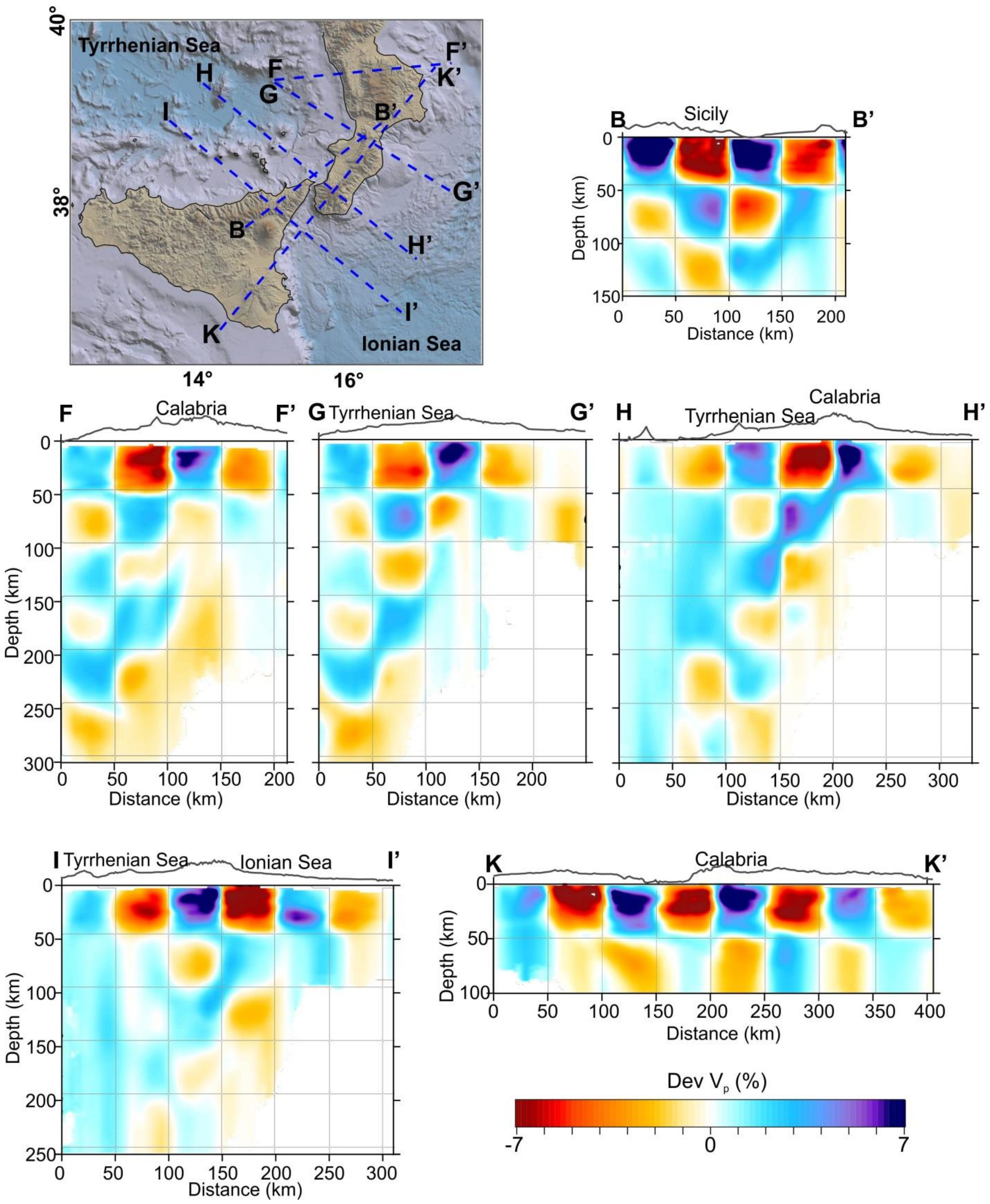

Figure S3. Results of the checkerboard test with initial models defined along several vertical sections. Grey thin lines highlight the configurations of the synthetic anomalies (50x50 km). The figure was obtained using the software Surfer (version 10.7.972; http://www.goldensoftware.com/products/surfer) and Corel Draw Graphics Suite (version X5; http://www.corel.com).

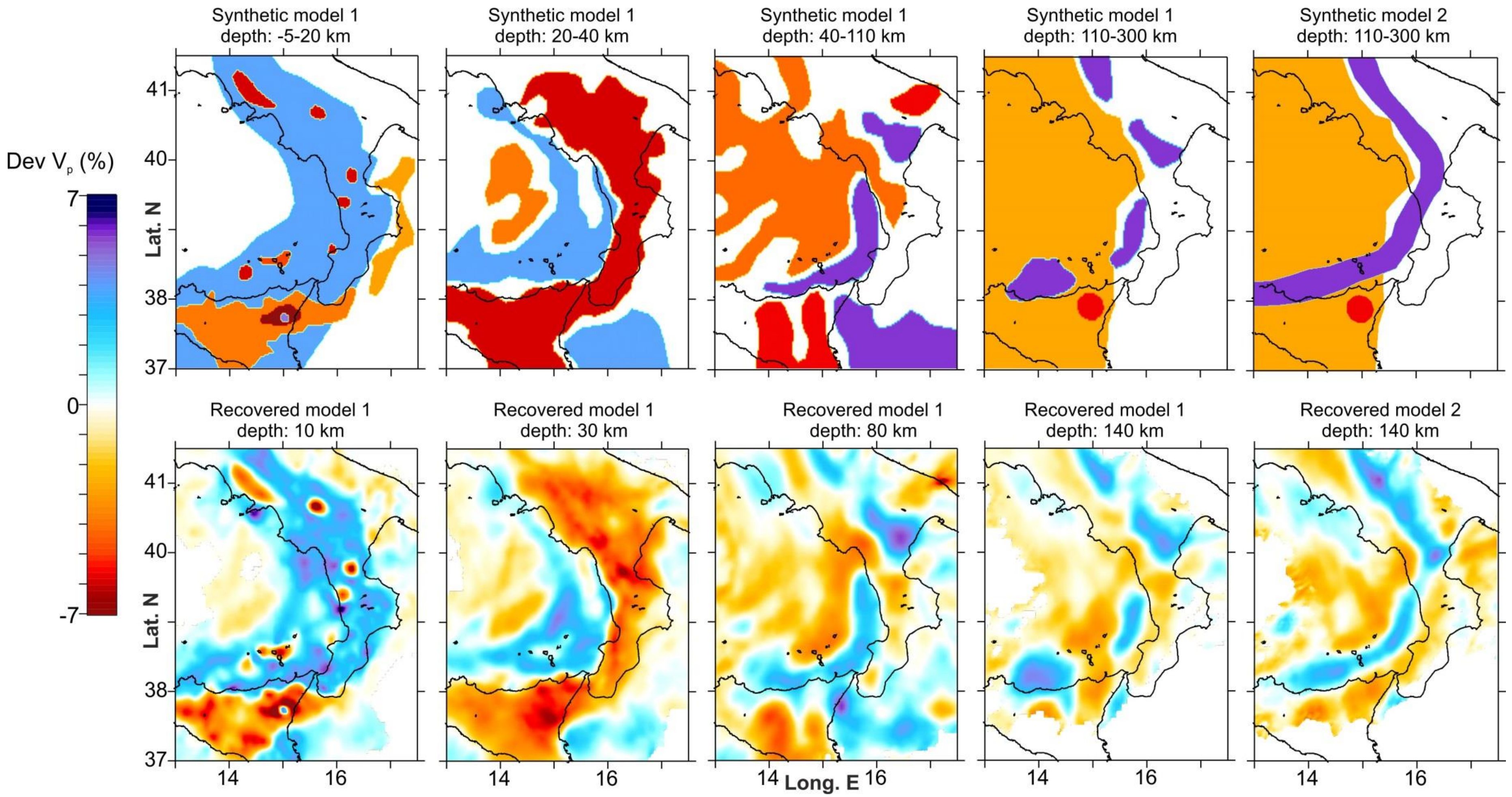

Figure S4. Synthetic tests with realistic anomalies: upper row is the initial model and lower row is the reconstruction result. Results for two models, which differ only at depth below 100 km, are presented in columns 1 to 4 for the 1st model, and in column 5 for the 2nd model. The figure was obtained using the software Surfer (version 10.7.972; http://www.goldensoftware.com/products/surfer) and Corel Draw Graphics Suite (version X5; http://www.corel.com).

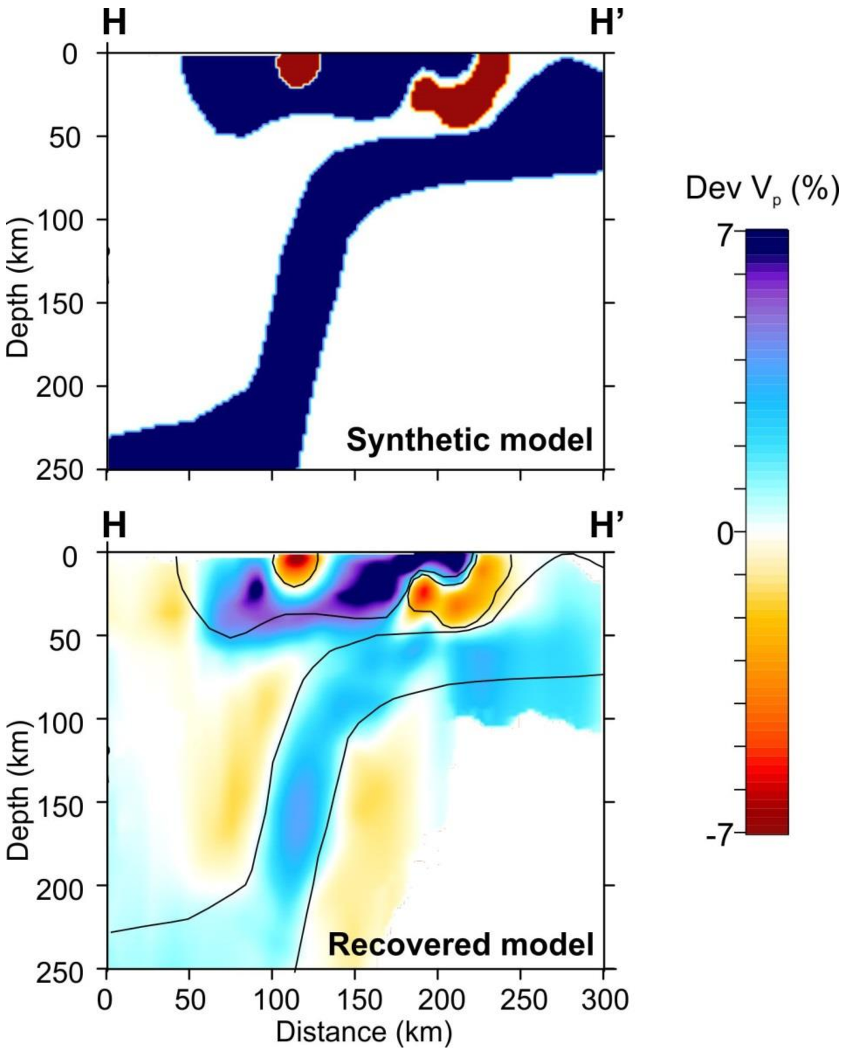

Figure S5. Synthetic test with the definition of a slab shape in the section HH'. Thin black lines indicate the shapes of the initial synthetic anomalies. The figure was obtained using the software Surfer (version 10.7.972; http://www.goldensoftware.com/products/surfer) and Corel Draw Graphics Suite (version X5; http://www.corel.com).
